# Modeling of Patient‐Derived 3D Organoids of Thyroid Cancer: From Cells to Care

**DOI:** 10.1155/ije/1021058

**Published:** 2026-08-06

**Authors:** Nam Kyung Kim, So Jeong Lee, Junu Kim, Jun Sung Lee, Hyeok Jun Yun, Seok-Mo Kim, Su-Jin Shin, Yong Sang Lee, Hang-Seok Chang

**Affiliations:** ^1^ Department of Surgery, Thyroid Cancer Center, Gangnam Severance Hospital, Institute of Refractory Thyroid Cancer, Yonsei University College of Medicine, Seoul, South Korea, yonsei.ac.kr; ^2^ Department of Pathology, Gangnam Severance Hospital, Yonsei University College of Medicine, Seoul, South Korea, yonsei.ac.kr

**Keywords:** anaplastic thyroid carcinoma, organoids, patient-derived organoid, radioiodine-refractory papillary thyroid carcinoma, thyroid cancer

## Abstract

**Introduction:**

Three‐dimensional (3D) organoids derived from patient tissues have emerged as promising preclinical models that preserve tumor architecture and phenotypic heterogeneity better than conventional two‐dimensional cultures. This study aimed to establish patient‐derived 3D organoid models for anaplastic thyroid carcinoma (ATC) and clinically radioiodine‐refractory papillary thyroid carcinoma (RAIR‐PTC) and evaluate whether they retained the histopathologic and immunophenotypic features of their source tumors.

**Methods:**

Tumor tissues from patients with ATC and RAIR‐PTC were collected, dissociated, and embedded in Matrigel for 3D culture. Organoid growth was monitored during serial passaging. Histopathologic, immunophenotypic, and targeted molecular analyses were performed.

**Results:**

The TCO001 sample, derived from ATC, showed substantial growth for more than five months, up to Passage 11, whereas the TCO002 sample, derived from RAIR‐PTC, exhibited limited proliferation and could not be maintained beyond Passage 3 after four months. These differences in culture performance were further supported by Ki‐67 and p53 staining, which demonstrated markedly higher proliferative activity and a more aberrant p53 expression pattern in TCO001 than in TCO002. Histopathologic and immunophenotypic analyses showed a dedifferentiated, ATC‐like phenotype in TCO001 and a more differentiated thyroid carcinoma‐like phenotype in TCO002. Targeted molecular analysis further supported the biological differences between the two lines: TCO001 harbored a BRAF mutation and a TERT promoter alteration, whereas TCO002 was wild type for both genes, consistent with the matched primary tumor tissue.

**Discussion:**

This study provides preliminary proof of concept that patient‐derived organoid technology can be extended to aggressive thyroid cancer phenotypes and preserve subtype‐relevant differences in differentiation state. Although preliminary, these findings support the value of thyroid cancer organoids as patient‐relevant preclinical models and provide a basis for future molecular, functional, and therapeutic studies in aggressive thyroid cancer.

## 1. Introduction

Thyroid cancer is one of the most common endocrine malignancies, and although most cases are well differentiated and have favorable outcomes, a subset of tumors shows aggressive clinical behavior and remains difficult to treat [1–4]. In particular, anaplastic thyroid carcinoma (ATC) is rare but highly lethal because of its rapid progression and resistance to conventional therapies, and some papillary thyroid carcinomas (PTCs) become refractory to standard treatment, including radioactive iodine, and thus pose an important clinical challenge [5–7].

Cancer research has historically relied on two‐dimensional (2D) cell cultures and *in vivo* models such as mice. Although those systems have provided important insights, they fail to replicate the three‐dimensional (3D) architecture and tumor heterogeneity observed in human cancers. Patient‐derived organoids have therefore emerged as promising preclinical models because they preserve key structural and phenotypic features of primary tumors and provide a relevant platform for studying tumor biology and therapeutic responses [8–11].

Progress in thyroid organoid culture systems has fallen behind that for other solid tumors, in part because thyroid follicular cells have a slow turnover rate [12]. Nevertheless, recent innovations have enabled the generation of thyroid organoids that can perform key functions, including thyroglobulin (TG) synthesis, iodide uptake, and thyroid hormone production [12]. These organoid models offer new opportunities to explore thyroid physiology, cancer progression, genetic alterations, and disease modeling. Building on that progress, recent studies have also demonstrated the feasibility of generating functional thyroid organoids and patient‐derived PTC organoids, supporting the potential of this platform for disease modeling and translational research [13, 14].

Nonetheless, evidence for organoid models derived from aggressive thyroid cancers remains insufficient. Accordingly, this study aimed to establish patient‐derived organoid models from ATC and clinically radioiodine‐refractory PTC (RAIR‐PTC) and evaluate whether they retained the histopathologic and immunophenotypic characteristics of their source tumors.

## 2. Materials and Methods

### 2.1. Key Resources and Materials

A comprehensive list of the reagents, materials, and media used in this study is provided in Table 1. The detailed composition of the organoid culture medium and the biological rationale for each major component are summarized in Supporting Table 1.

**TABLE 1:** List of reagents, materials, and media.

Reagents	Materials	Media
· GlutaMAX Supplement (Gibco, USA, #35050–061)	· Biological samples: fresh human ATC and PTC tissue samples from surgery at the Thyroid Cancer Center, Gangnam Severance Hospital, Yonsei University College of Medicine, South Korea	1. Dissociation medium for tumor tissue
· HEPES buffer solution (Sigma, USA, #83264)	· 48‐well cell culture plate (SPL, South Korea, #30048)	· 15 mL of PBS with 2% AA
· Penicillin–streptomycin (10,000 U/mL) (Gibco, USA, #15140–122)	· 3‐mL transfer pipette (Sigma, South Korea, #LT.TP–3 M)	· 5 mL of collagenase IV (2 mg/mL)
· Antibiotic–antimycotic (100x) (Gibco, USA, #15240–062)	· Pasteur pipettes (KOREAACE, South Korea, #LT.PP230)	2. Organoid culture medium
· B‐27 TM supplement (50X), minus vitamin A (Gibco, USA, #12587–010)	· 15‐mL conical centrifuge tube (SPL, South Korea, #50015)	· 50 mL Advanced DMEM/F12+++(1x): 500 mL of Advanced DMEM/F12 supplemented with 10 mL of penicillin–streptomycin (10,000 U/mL), 5 mL of GlutaMAX supplement (100x), and 5 mL of HEPES (1 M).
· N‐Acetyl‐L‐cysteine (Sigma, USA, #A7250)	· 50‐mL conical centrifuge tube (SPL, South Korea, #50050)	· 1 mL B‐27 TM supplement, minus vitamin A (50x)
· Recombinant murine EGF (PeproTech, USA, #315–09)	· 5‐mL serological pipette (Thermo Fisher, USA, #170355 T)	· 100 μL N‐Acetyl‐L‐cysteine (1.25 M)
· Recombinant human FGF10 (PeproTech, USA, #100–26)	· 10‐mL serological pipette (Thermo Fisher, USA, #170356T	· 25 μL recombinant murine EGF (100 μg/mL)
· Recombinant human R‐spondin‐1 (PeproTech, USA, #120–38)	· 25‐mL serological pipette (Thermo Fisher, USA, #170357 T)	· 100 μL recombinant human FGF10 (50 μg/mL)
· Recombinant human Noggin (PeproTech, USA, #120–10 C)	· Cell culture dish, 60 mm (Eppendorf, Germany, #30701119)	· 100 μL recombinant human R‐spondin‐1 (100 μg/mL)
· Wnt‐3a, human recombinant (Biovision, USA #P1194)	· 1.7‐mL MaxyClear Snaplock microcentrifuge tube (Axygen, USA, #MCT‐175‐C)	· 50 μL recombinant human Noggin (100 μg/mL)
· Thyrotropic hormone from the human pituitary (Sigma, USA, #T9265)	· Cryogenic vials (Thermo Fisher, USA, #368632)	· 30 μL Wnt‐3a, human recombinant (50 μg/mL)
· PBS without calcium and magnesium (Cytiva, USA, #SH30256.01)		· 100 μL thyrotropic hormone from human pituitary (155 mIU/mL)
· Collagenase from Clostridium histolyticum, Type IV (Sigma, USA, #C5138)		
· Matrigel growth factor reduced (GFR) basement membrane matrix, phenol red–free, LDEV‐free (Corning, USA, #356231)		
· Recovery cell culture freezing medium (Gibco, USA, #12648–010)		

### 2.2. Patients and Tissue Specimens

Cancer tissues were collected from patients with histologically confirmed ATC or PTC treated at the Thyroid Cancer Center, Gangnam Severance Hospital, Yonsei University College of Medicine (Table 2). For TCO001, only a limited amount of tumor tissue was obtained during emergency airway management and tracheostomy; therefore, no separate matched primary‐tissue pathology report or immunohistochemical evaluation was available. For TCO002, although the histopathologic diagnosis was PTC, the patient showed clinically radioiodine‐refractory disease behavior. After bilateral total thyroidectomy with central compartment neck dissection and bilateral modified radical neck dissection, the patient received three courses of high‐dose radioactive iodine therapy (250 mCi each; cumulative dose, 750 mCi). Despite those treatments, follow‐up imaging demonstrated recurrent metastatic lymph nodes in the neck and metastatic lung nodules, so systemic therapy with lenvatinib was initiated. Matched immunohistochemical evaluation of the archival primary tumor tissue had not been performed.

**TABLE 2:** Sample list of study subjects and organoid culture outcomes.

	TCO001	TCO002
*Clinical and pathological features*		
Sex/age	M/59	F/55

Date of tissue sampling (operation date)	2020‐05‐27	2020‐11‐03

Operation	Excision of lesion of the thyroid and tracheostomy^∗^	BTT with CCND and MRND, Bil.

Diagnosis	Anaplastic thyroid carcinoma	Papillary thyroid carcinoma

Additional pathology report	Not available^∗^	· Histologic subtype: conventional
		· Maximum diameter of tumor (location): 6.5 cm (both lobes, upper to lower pole)
		· Gross capsular involvement, microscopic perithyroidal soft tissue involvement
		· Lymphovascular invasion: present, extensive
		· Lymph node, total (29/59): metastatic carcinoma (maximal diameter: 2.2 cm) with perinodal soft tissue extension

Primary‐tissue molecular findings	Not available^∗^	·BRAF wild type
		·TERT wild type

Clinical disease course		· Clinically radioiodine‐refractory disease behavior
		· 3 courses of high‐dose radioactive iodine therapy (250 mCi each; cumulative dose, 750 mCi)
		· Recurrent metastatic cervical lymph nodes and metastatic lung nodules
		· Lenvatinib initiated

*Organoid culture outcomes*		
Result of cell culture	· Passage 11 culture completed	· Passage 3 culture completed
	· 10 vials of cell stock produced	· 7 vials of cell stock produced

Organoid molecular findings	· BRAF mutation	· BRAF wild type
	· TERT promoter C228T mutation	· TERT wild type

*Note:* Bil, bilateral.

Abbreviations: BTT, bilateral total thyroidectomy; CCND, central compartment neck dissection; MRND, modified radical neck dissection; TCO, thyroid cancer organoid.

^∗^Considering the patient’s overall clinical status and future treatment plan, we determined that tissue excision for pathological confirmation and a tracheostomy were more appropriate than radical surgery.

The resected tumor tissues were isolated and stored separately in phosphate‐buffered saline (PBS) buffer containing 2% antibiotic–antimycotic (AA).

The study was conducted in accordance with the Declaration of Helsinki (as revised in 2013). Human participation was reviewed and approved by the Institutional Review Board of Gangnam Severance Hospital, Yonsei University College of Medicine, Seoul, South Korea (Approval No. 3‐2020‐0293). The patients/participants provided written informed consent to participate in this study.

### 2.3. Organoid Primary Culture

Tumor tissues were rinsed twice with PBS buffer containing 2% AA. Then, they were minced in a tube with a dissociation medium containing 20 mL of PBS buffer with 2% AA and 0.5 mg/mL collagenase Type IV. The minced tumor cells were incubated in a shaking incubator at 37°C for 2 h and then centrifuged at 800 × g for 5 min. The supernatant was removed, and the cells were resuspended in 5 mL of PBS buffer containing 2% AA. The suspension was left to settle until all the cell debris sank to the bottom, after which the buffer was transferred to a new 15‐mL tube and centrifuged again at 800 × g for 5 min. The supernatant was removed, and the cells were resuspended in 1 mL of PBS buffer containing 2% AA, allowed to settle, and centrifuged one more time at 800 × g for 5 min. Then, the supernatant was removed.

Matrigel was prepared on ice, and the cell pellet was resuspended in 400 μL of Matrigel.

Using a 48‐well plate prewarmed in a 37°C incubator, 25 μL of the cell–Matrigel suspension was quickly dispensed into each well. Cells were not loaded into the edge wells of the 48‐well plate to prevent the Matrigel from drying. Once the plate was loaded with cells, it was placed in a 37°C incubator for 10 min to solidify the Matrigel.

Next, 350 μL of preprepared organoid culture medium at room temperature was added to each well. The organoid culture medium used in this study was based on the thyroid organoid culture system described by Saito et al. [12], with the final concentrations of individual components summarized in Supporting Table 1. TSH and Wnt‐3a were retained because both are components of the established thyroid organoid medium, and previous thyroid organoid studies showed that TSH increased organoid number and cell count in a concentration‐dependent manner. The cell status was checked under a microscope, and then, the cells were cultured overnight in a 37°C incubator. The next day, the cell status was assessed under a microscope. The culture medium was replaced every 2–3 days to monitor cell growth.

After the organoid primary culture, cell growth was monitored under a microscope, and subculture was performed when the cells occupied more than 80% of the Matrigel.

To subculture the cells, the medium was removed from the 48‐well plate, which was then washed twice with 500 μL of 1x PBS solution. Then, 1 mL of 1x PBS solution was added to each well containing organoid cells, and the Matrigel was broken into small pieces by pipetting. The broken Matrigel was transferred to a 1.5‐mL tube and centrifuged at 800 × g, 4°C, for 5 min. The supernatant was removed, 1 mL of 1x PBS solution was added, and the solution was vortexed to disperse the cell pellet. The vortexed solution was centrifuged at 800 × g, 4°C, for 5 min, and the supernatant was removed. Next, 300 μL of Matrigel was added and mixed well with the cell pellet by pipetting, and 25 μL of the solution per well was dispensed into a 48‐well plate prepared in a 37°C incubator the day before. The seeded cells were placed in a 37°C incubator for 10 min to solidify the Matrigel. After that, 350 μL of the medium that had previously been removed at room temperature was added to each well. The cell status was assessed under a microscope, and the plate was incubated at 37°C for 24 h. The next day, organoid growth was evaluated under a microscope.

### 2.4. Histopathologic, Immunophenotypic, and Targeted Molecular Evaluation of Organoids

To evaluate whether the established organoid lines retained the histopathologic and lineage‐related features of the source tumors, organoid cell blocks were prepared from serially cultured organoids. Representative passages were selected from each organoid line, including multiple passages from the line with long‐term expansion, to assess phenotypic reproducibility over time. Formalin‐fixed, paraffin‐embedded cell‐block sections were subjected to hematoxylin and eosin (H&E) staining and immunostaining. In the absence of matched primary‐tissue immunohistochemical analysis for either specimen, organoid morphology and immunophenotype were interpreted in the context of the known clinicopathologic diagnosis.

The immunostaining panel was selected to assess epithelial identity, thyroid follicular lineage, degree of retained thyroid differentiation, and major differential diagnostic considerations. The primary markers of interest for distinguishing differentiated thyroid carcinoma (DTC)‐like from ATC‐like phenotypes were TTF‐1, PAX8, and TG. CK was used to confirm epithelial differentiation. CD45, synaptophysin, CD5, and calcitonin staining were performed to exclude lymphoid contamination and alternative lines of differentiation. Additional Ki‐67 and p53 staining was performed to assess proliferative activity and biological aggressiveness.

Sections from the formalin‐fixed, paraffin‐embedded cell blocks were immunostained using a Ventana Benchmark XT automated staining system (Ventana Medical Systems, Tucson, AZ, USA) according to the manufacturer’s protocol. The following primary antibodies were used for immunohistochemistry: TTF‐1 (clone 8G7G3/1; dilution 1:150; Dako, Carpinteria, CA, USA), PAX8 (clone MRQ‐50; dilution 1:100; Cell Marque, Rocklin, CA, USA), TG (clone 2H11 + 6E1; RTU; Cell Marque), CK (clone AE1/AE3; dilution 1:500; Novocastra, Leica Biosystems, Newcastle, UK), CD45 (clone 2B11 + PD7/26; dilution 1:100; Dako), synaptophysin (clone DAK‐SYNAP; dilution 1:100; Dako), CD5 (clone 4C7; dilution 1:25; Novocastra, Leica Biosystems), calcitonin (polyclonal; RTU; Cell Marque), Ki‐67 (clone MIB‐1; dilution 1:800; Dako), and p53 (clone NCL‐p53‐DO7; dilution 1:2500; Novocastra).

TTF‐1 and PAX8 were interpreted as positive only when nuclear staining was identified. Weak cytoplasmic TTF‐1 staining without convincing nuclear reactivity was cautiously interpreted as nonspecific staining, particularly in cell block–based preparations. TG was interpreted as a marker of retained thyroid follicular differentiation.

Staining patterns were compared between the two organoid lines and, when available, across serial passages to assess phenotypic reproducibility.

Targeted molecular analysis of organoid DNA was performed to assess selected driver alterations. BRAF mutation analysis was conducted using peptide nucleic acid clamping‐based real‐time polymerase chain reaction, and TERT promoter mutation analysis was conducted using pyrosequencing. For TCO002, molecular results for BRAF and TERT were available from the corresponding primary tumor tissue and were compared with the organoid findings.

## 3. Results

### 3.1. Results of Organoid Cell Culture

Single thyroid cells obtained from ATC and RAIR‐PTC tissues were cultured in a 3D environment to establish thyroid organoids. The cells were embedded in Matrigel and incubated in an organoid culture medium containing growth factors, including epidermal growth factor (EGF), fibroblast growth factor‐10 (FGF‐10), R‐spondin‐1, Noggin, Wnt‐3a, and thyroid‐stimulating hormone (TSH).

During the early stages of culture, the samples, TCO001 and TCO002, showed similar initial growth patterns. The cells initially appeared as small spots within the Matrigel matrix and then gradually expanded into larger clusters, forming organoid‐like structures. This growth pattern mirrors the cellular heterogeneity and structural complexity typically observed in in vivo tissues. However, significant differences in long‐term growth between the two samples were observed.

### 3.2. TCO001 Sample: Substantial Growth and Long‐Term Culturing

**FIGURE 1:**
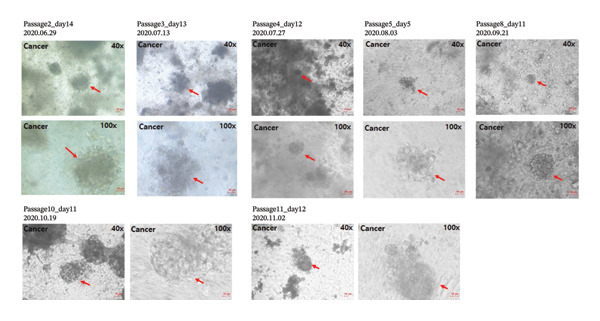
TCO001. Representative images from selected serial passages of TCO001 are shown. The TCO001 sample, derived from anaplastic thyroid cancer, showed rapid and consistent growth and was successfully subcultured up to Passage 11 over five months, producing 10 vials of cell stock. The formation and morphology of organoid structures were observed at each passage stage. On Day 14 of Passage 2, the organoids exhibited a relatively well‐defined spherical structure. However, as the passages progressed, the organoids began to increase in morphological diversity, deviating from the initial spherical shape and forming irregular structures. This phenomenon was observed from Day 13 of Passage 3 to Day 12 of Passage 11, during which the organoids exhibited various sizes and morphological heterogeneity. The arrows marked in the magnified images indicate the same cell, highlighting the morphological changes over time.

The TCO001 sample, derived from an ATC, showed rapid and consistent growth throughout the passages. After an initial primary culture period of five days, the cells were subcultured at Passage 1. During a period of five months, TCO001 was successfully subcultured up to Passage 11, with 10 vials of cell stock produced, demonstrating a strong capacity for long‐term growth and proliferation. The organoid structures in TCO001 grew progressively larger with each passage, maintaining their integrity and 3D architecture (Table 2, Figure 1).

### 3.3. TCO002 Sample: Limited Growth and Early Culture Cessation

**FIGURE 2:**
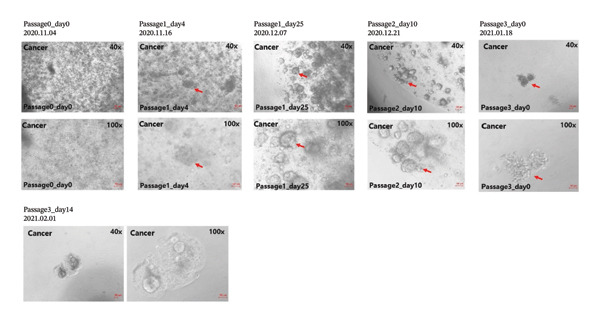
TCO002. Representative images from selected serial passages of TCO002 are shown. The TCO002 sample, derived from RAIR‐PTC, exhibited slow growth and was subcultured up to Passage 3 over 4 months, producing seven vials of cell stock. In the early passages, the structures appeared irregular, and distinct spherical formations were not observed. However, as the passage stage culture progressed, organoids with spherical structures and those with irregular morphologies were observed together. The arrows in the magnified images indicate the same cell, highlighting the morphological changes over time.

In contrast, the TCO002 sample, derived from RAIR‐PTC, showed a slower initial growth rate. The TCO002 sample required a primary culture period of 10 days before subculturing at Passage 1. Despite successful initial subculturing, the sample could only be subcultured up to Passage 3 over a four‐month period, producing 7 vials of cell stock, after which cell proliferation slowed considerably, and the culture was terminated due to limited cell growth (Table 2, Figure 2).

### 3.4. Histopathologic, Immunophenotypic, and Targeted Molecular Characterization of Thyroid Cancer Organoids

In the H&E‐stained samples, both organoid lines formed malignant epithelial cell clusters, but their morphologic appearances differed along a spectrum of thyroid cancer differentiation. TCO002 showed a morphology more reminiscent of differentiated PTC, with more cohesive cell clusters and relatively rounder nuclei, whereas TCO001 showed greater nuclear pleomorphism and more bizarre nuclear features, suggesting a more dedifferentiated, ATC‐like morphology. However, morphology alone is insufficient to definitively distinguish DTC from ATC; therefore, lineage‐ and differentiation‐related markers were evaluated.

CK staining was positive in both TCO001 and TCO002, confirming that both organoid lines were composed of epithelial tumor cells. Among the thyroid differentiation markers, the clearest distinction between the two lines was observed in TTF‐1 and TG expression. TCO002 showed nuclear TTF‐1 positivity and retained TG expression, supporting the preservation of thyroid follicular differentiation. In contrast, TCO001 lacked convincing nuclear TTF‐1 staining and was negative for TG, consistent with marked dedifferentiation. Weak cytoplasmic TTF‐1 staining was observed in some preparations, but this was cautiously interpreted as nonspecific reactivity because true TTF‐1 positivity should be nuclear. PAX8 showed nuclear positivity in both TCO001 and TCO002, supporting preservation of the thyroid follicular epithelial lineage in both organoid lines.

Taken together, these findings indicate that both TCO001 and TCO002 retained epithelial and thyroid‐lineage features, but they differed in the degree of preserved thyroid differentiation. TCO001 showed a dedifferentiated, ATC‐like immunophenotype characterized by the preservation of thyroid origin and loss of follicular differentiation markers, whereas TCO002 retained an immunophenotype consistent with DTC.

Additional immunostaining was performed mainly for differential diagnostic purposes. Both lines were negative for CD45, arguing against significant lymphoid contamination. Synaptophysin, CD5, and calcitonin were also negative, making alternative neuroendocrine or nonfollicular thyroid differentiation unlikely.

The staining pattern was reproducible across serial passages in both organoid lines. TCO001 consistently retained a dedifferentiated profile characterized by preserved CK and PAX8 expression with the loss of convincing nuclear TTF‐1 and largely absent TG, whereas TCO002 maintained a differentiated profile with CK, nuclear PAX8, nuclear TTF‐1, and focal TG expression. These findings suggest that lineage‐ and differentiation‐related phenotypic characteristics were maintained during in vitro expansion.

**FIGURE 3:**
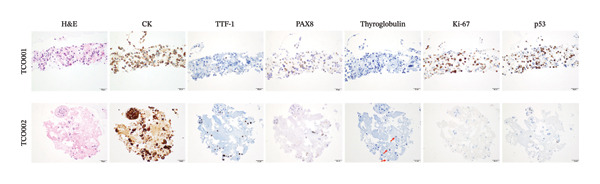
Histopathologic, immunophenotypic, and additional biological characterization of thyroid cancer organoids. Representative images of H&E and immunostaining of organoid cell blocks from TCO001 and TCO002 are shown in the following order: H&E, CK, TTF‐1, PAX8, thyroglobulin, Ki‐67, and p53. On H&E, TCO001 showed a less differentiated appearance with more marked nuclear atypia, whereas TCO002 showed a morphology more reminiscent of papillary thyroid carcinoma. Both organoid lines were positive for CK and showed nuclear PAX8 positivity. In contrast, TCO001 lacked convincing nuclear TTF‐1 staining and was negative for thyroglobulin, whereas TCO002 retained nuclear TTF‐1 and thyroglobulin expression. Additional markers of tumor behavior also differed between the two lines. TCO001 showed a markedly higher Ki‐67 labeling index (approximately 80%–90%) and diffuse strong nuclear p53 staining, whereas TCO002 showed a very low Ki‐67 index (< 1%) and limited nuclear p53 staining. Original magnification, × 400.

Additional markers of biological aggressiveness also differed between the two organoid lines. TCO001 showed a markedly higher Ki‐67 labeling index than TCO002 (approximately 80%–90% vs. < 1%), indicating substantially greater proliferative activity. In addition, TCO001 showed diffuse, strong nuclear p53 staining, whereas TCO002 showed limited nuclear p53 staining (Figure 3).

Targeted molecular analysis further demonstrated a BRAF mutation and a TERT promoter alteration in TCO001, whereas TCO002 was wild type for both BRAF and TERT. In TCO002, the organoid findings were concordant with the available BRAF and TERT results from the matched primary tumor tissue.

## 4. Discussion

Thyroid cancer generally carries a favorable prognosis, particularly in well‐differentiated subtypes such as PTC and follicular thyroid carcinoma [13, 15]. However, aggressive tumors, including ATC and refractory thyroid cancers, remain major therapeutic challenges due to rapid progression or resistance to standard treatments [16, 17]. The poor outcomes of these advanced thyroid cancers highlight the need for informative preclinical models that can adequately represent aggressive disease biology [15]. In this context, patient‐derived 3D organoids have emerged as a promising platform for studying tumor behavior, phenotypic heterogeneity, and therapeutic response [10, 15, 18].

Despite the considerable promise of thyroid cancer organoid cultures, establishing and sustaining them long‐term remains technically challenging. These systems require highly specific growth conditions, including a precisely regulated balance of key growth factors such as EGF, FGF10, R‐spondin‐1, and TSH, and their long‐term success likely depends not only on technical optimization but also on the intrinsic biological properties of the tumor [12, 14, 15, 19].

In this study, we established patient‐derived thyroid cancer organoid cultures from two clinically challenging thyroid tumors and observed distinct growth patterns and differentiation states during serial passaging. Although the sample size was small, our findings provide proof of concept that patient‐derived organoid models can be generated from biologically aggressive thyroid cancers.

Notably, TCO001 was successfully cultured for more than five months and expanded to Passage 11, whereas TCO002 showed more limited expansion and could not be maintained beyond early passages. These markedly different growth kinetics likely reflect biologically meaningful tumor‐to‐tumor heterogeneity [20]. The prolonged growth of TCO001 suggests that some aggressive thyroid tumors can harbor cell populations that adapt relatively well to ex vivo 3D culture conditions [19]. This finding is notable given the historically slow progress in thyroid organoid culture, likely related in part to the slow turnover rate of thyroid follicular cells. In contrast, the more limited expansion of TCO002 could reflect reduced proliferative capacity, lower abundance of growth‐competent tumor cells, or other intrinsic biological differences in the source tumor [14]. Although these possibilities remain speculative in the absence of molecular and functional analyses, the observed differences suggest that culture behavior itself can reflect tumor‐specific biology.

Additional markers of tumor behavior further reinforced the biological distinction between the two organoid lines. The markedly higher Ki‐67 labeling index in TCO001 was consistent with its more sustained growth during serial culture, whereas the very low proliferative index in TCO002 paralleled its limited expansion potential. Similarly, the diffuse, strong p53 staining in TCO001 was interpreted as an aberrant expression pattern supporting a more dedifferentiated and aggressive phenotype.

Targeted molecular analysis provided further support for this distinction, showing a BRAF mutation and a TERT promoter alteration in TCO001, whereas TCO002 was wild type for both genes. In TCO002, the organoid findings were concordant with the available BRAF and TERT results from the matched primary tumor tissue. Although this limited analysis does not establish full genomic fidelity, it provides partial molecular evidence that the organoid retained clinically relevant features of the matched RAIR‐PTC tumor.

A major strength of this study is the addition of histopathologic and immunophenotypic validation, which strengthened the biologic interpretation of the organoid models beyond simple morphologic observation. Although the H&E findings suggested a differentiated appearance in TCO002 and an anaplastic phenotype in TCO001, morphology alone is insufficient to definitively distinguish DTC from ATC in organoid preparations [13, 15]. For this reason, the immunophenotypic analysis was essential for validating the biological identity of the organoid cultures [13, 21].

Nuclear PAX8 positivity in both lines supports the preservation of thyroid follicular epithelial origin, whereas TTF‐1 and TG more clearly reflect the degree of retained follicular differentiation [19, 21, 22]. The weak cytoplasmic TTF‐1 staining observed in TCO001 underscores an important technical point: In cell block–based preparations, true positivity should be defined strictly by nuclear staining to prevent the overinterpretation of nonspecific reactivity [19]. Ancillary markers were overall supportive of epithelial thyroid tumor identity and helped exclude major mimickers [14, 19]. Together, these findings strengthen the interpretation that TCO001 and TCO002 were not merely nonspecific 3D aggregates, but organoid cultures that retained clinically meaningful differences in their thyroid cancer differentiation states.

Another important finding is the reproducibility of the immunophenotypic pattern across serial passages. The overall staining profile remained stable during culture, suggesting that lineage‐ and differentiation‐related phenotypic characteristics were maintained during in vitro expansion [19]. This phenotypic stability is an important requirement for any in vitro tumor model intended for biological investigation, translational research, or future therapeutic testing.

Our findings should be interpreted in the context of the current thyroid organoid literature. Most previously published thyroid organoid studies have focused on differentiated PTC. Yang et al. established PTC organoid lines from clinical specimens with a reported success rate of 77.6%, demonstrating serial passaging, cryopreservation, and the preservation of histologic and genomic features [14]. Similarly, Chen et al. reported long‐term patient‐derived PTC organoids that retained their histopathologic profiles and mutational landscapes and were used in drug–response studies, including BRAF inhibitor–based combination therapies [13]. In addition, patient‐derived PTC organoids were developed for radioactive iodine–refractory screening, and more recent differentiated thyroid cancer organoid models have been applied to functional radioiodine uptake assays [21, 22]. Together, those studies established the feasibility and translational value of thyroid organoids, but their emphasis remained largely on differentiated PTC, rather than on highly aggressive thyroid cancer phenotypes.

In that context, the novelty of our study lies less in large‐scale organoid establishment and more in the biological spectrum represented by the two tumors analyzed. Recent reviews have emphasized that the evidence for organoid models derived from aggressive thyroid cancers remains insufficient and that models of advanced and dedifferentiated thyroid carcinoma are an important unmet need in the field [15]. Our study directly addresses that gap by demonstrating that organoids established from ATC and RAIR‐PTC can preserve distinct differentiation‐related phenotypes during serial culture. Thus, unlike most prior thyroid organoid studies, which have focused on differentiated PTC, our study provides preliminary evidence that patient‐derived organoid technology can be extended to aggressive thyroid cancer phenotypes while preserving subtype‐relevant differences in differentiation state.

This study has several limitations. First, the sample size was limited to two cases, and the findings should therefore be interpreted as a preliminary proof of concept rather than as generalized clinical evidence. This small number of cases reflects our primary aim in this study, which was to successfully establish organoids from aggressive thyroid cancer specimens. Second, quantitative growth parameters, such as organoid size and formation efficiency, were not systematically recorded, limiting detailed comparisons of culture performance between the two lines. Third, because this was a case‐based proof‐of‐concept study of aggressive thyroid cancer organoid establishment, we did not perform systematic factor‐withdrawal experiments to determine the individual contributions of Wnt‐3a, TSH, and other medium components. Therefore, although the medium composition was based on previous thyroid organoid studies and is now described more clearly, the specific role of each component in organoid growth and differentiation could not be determined. Fourth, matched primary‐tissue immunohistochemical data were unavailable for either case, limiting direct pathological comparison between the organoids and their source tumors. Although the H&E, immunophenotypic, and limited targeted molecular analyses substantially strengthened our pathologic validation, we did not perform comprehensive genomic profiling, transcriptomic analysis, or functional drug–response testing in these lines. As a result, we cannot yet determine whether these organoids fully retain the broader molecular landscape of the parental tumors or predict the treatment response [13]. Fifth, the current marker panel was designed primarily to confirm epithelial identity, establish thyroid lineage, and compare differentiation status, rather than to comprehensively define the molecular evolution of aggressive thyroid cancer [15].

Future studies should include additional cases, standardized quantitative growth metrics, molecular characterization of matched tumors and organoids, and functional testing to better define the translational potential of thyroid cancer organoids. The incorporation of stromal or immune coculture systems could further improve the biological relevance of these models, particularly for aggressive thyroid cancers in which tumor–microenvironment interactions are likely to contribute to therapeutic resistance [15]. In this regard, future platforms that integrate peripheral blood mononuclear cell coculture or immune checkpoint–based assays, including anti‐PD‐1 response models, could help to evaluate patient‐specific tumor–immune interactions in vitro [23, 24]. In parallel, technological refinement using microfluidic devices or air–liquid interface systems could support more complex and physiologically relevant culture conditions, thereby expanding the utility of thyroid cancer organoids for drug testing and mechanistic studies. Although such applications remain beyond the scope of this proof‐of‐concept study, they are important future directions toward more informative preclinical platforms and, ultimately, more individualized therapeutic strategies.

## 5. Conclusions

In conclusion, although the establishment of patient‐derived 3D thyroid cancer organoids remains technically challenging, our findings provide preliminary evidence that organoid technology can be extended beyond differentiated thyroid carcinoma to aggressive thyroid cancer phenotypes. Compared with conventional 2D cultures, these models provide a more representative platform for investigating aggressive thyroid tumor biology and may support the future development of more precise therapeutic strategies while reducing ineffective or unnecessary interventions. The successful establishment of these organoid models therefore represents an important step toward translational and potentially patient‐tailored applications in aggressive thyroid cancer.

## Author Contributions

Nam Kyung Kim, So Jeong Lee, Hyeok Jun Yun, Seok‐Mo Kim, Su‐Jin Shin, Yong Sang Lee, and Hang‐Seok Chang contributed to the conception and design of the study. Nam Kyung Kim and So Jeong Lee performed research and collected data. Nam Kyung Kim, So Jeong Lee, Hyeok Jun Yun, Seok‐Mo Kim, Su‐Jin Shin, and Yong Sang Lee analyzed and interpreted the data. Nam Kyung Kim and So Jeong Lee wrote the manuscript.

## Funding

This work was supported by the Korean Thyroid Association Outstanding Investigator Award 2022.

## Disclosure

All authors have critically revised the manuscript and read and approved the final version. A previous version of this manuscript was presented in abstract form at the 11th Conference of the European Society of Endocrine Surgeons (ESES) held in Izmir, Turkey, on May 22–24, 2025.

## Conflicts of Interest

The authors declare no conflicts of interest.

## Supporting Information

Additional supporting information can be found online in the Supporting Information section.

## Supporting information


**Supporting Information** Supporting Table 1 summarizes the composition of the organoid culture medium used in this study, including the final concentrations and biological rationale for each major component.

## Data Availability

The datasets used and/or analyzed during the current study are available from the corresponding author upon reasonable request.
