# Supplementary material for: Modeling of Patient‐Derived 3D Organoids of Thyroid Cancer: From Cells to Care
Source: Int J Endocrinol. 2026 Aug 6;2026:1021058. doi: 10.1155/ije/1021058 (PMC13445780; doi:10.1155/ije/1021058)
Supplement: Supplementary file 1 [file IJE-2026-1021058-s001.docx]

**Supplementary Table 1. Composition of the organoid culture medium used in this study and the biological rationale for each major component**

| Component | Final concentration used in this study | Biological rationale |
| --- | --- | --- |
| B27 supplement minus vitamin A | 1× | Standard supplement for long-term organoid maintenance |
| N-acetyl-L-cysteine | 2.5 mM | Antioxidant support for organoid survival and maintenance |
| EGF | 50 ng/mL | Supports epithelial and organoid growth |
| FGF10 | 100 ng/mL | Supports organoid expansion |
| R-spondin-1 | 200 ng/mL | Included in the established thyroid organoid medium formulation |
| Noggin | 100 ng/mL | Included in the established thyroid organoid medium formulation |
| Wnt-3a | 30 ng/mL | Included in the established thyroid organoid medium formulation |
| TSH | 0.31 mIU/mL | Thyroid-specific trophic factor retained based on previous thyroid organoid studies showing concentration-dependent increases in organoid number and cell count |
| GlutaMAX | 2 mM | Metabolic support for organoid culture |
| HEPES | 10 mM | Buffering support for organoid culture |
| Penicillin-streptomycin | 1× | Antimicrobial support for culture maintenance |

The medium composition was based on the thyroid organoid culture system previously reported by Saito et al.; the final concentrations used in this study are listed above.
